# Quantification of Total Porosity from CT Images by Segmenting Unhydrated Cement: A Model-Informed Framework Integrating POWERS’ Volume Model

**DOI:** 10.3390/ma19040686

**Published:** 2026-02-11

**Authors:** Haoran Liu, Eryu Zhu, Min Ji, Zhengwei Bai, Teng Li

**Affiliations:** 1 School of Civil Engineering, Beijing Jiao Tong University, Beijing 100044, China; 2 China Academy of Transportation Sciences, Beijing 100029, China

**Keywords:** cement paste, total porosity, X-ray computed tomography, powers’ hydration model, microstructure

## Abstract

Quantification of total porosity, including the nano-scale fraction, is critical for predicting the performance of cement-based materials but remains a significant challenge. While X-ray computed tomography (CT) is a powerful non-destructive tool, a fundamental trade-off between resolution and representative sample volume prevents the direct segmentation of nano-scale pores in macroscopically relevant specimens. Herein, we propose and validate a novel model-informed framework that overcomes this limitation by integrating the classical Powers’ hydration model with micro-CT analysis. The method circumvents the need for nano-scale resolution by deriving the total porosity from the volume fraction of the easily segmentable, micron-scale unhydrated cement phase. The framework’s validity was demonstrated by showing a strong correlation between the CT-derived total porosity and established porosity–strength relationships. Quantitative analysis indicated that the total porosity of the cement pastes ranged from 36.5% to 60.3% as the *w*/*c* ratio increased from 0.4 to 0.7. Laboratory strength data show good correlation (R^2^ > 0.98) with four porosity–strength prediction models, demonstrating the feasibility of applying the Powers’ volume model in CT-based analyses of cement pastes. This work transforms micro-CT from a qualitative imaging tool into a comprehensive technique for the quantitative microstructural characterization of cementitious materials.

## 1. Introduction

Cement-based materials are foundational to modern infrastructure, and their mechanical properties and durability are fundamentally governed by the evolution of their internal pore structure during hydration [1,2]. The Powers and Brownyard model [3] provides the classical theoretical framework for understanding these relationships, linking the water/cement (*w*/*c*) ratio and degree of hydration to the volume fractions of solid phases and porosity. Quantifying the total porosity, which includes both micron-scale voids and nano-scale capillary and gel pores, is therefore of paramount importance for performance prediction and material design [4,5,6,7]. The Powers and Brownyard model [3], developed in the 1940s, serves as the cornerstone of modern cement science. Based on the stoichiometry of hydration reactions, this model quantitatively establishes the volumetric relationships between the initial reactants (cement and water) and the resulting phases (hydration products, gel water, and capillary pores) as a function of the *w*/*c* ratio and the degree of hydration. Its primary feature lies in treating the hardened cement paste as a composite of unhydrated cement, solid hydration products (gel solids), and pores, allowing for the precise calculation of the volume fraction of each phase. Regarding applications, the model is extensively utilized not only to predict total porosity but also to estimate mechanical strength (via the gel/space ratio theory) and assess durability parameters such as permeability. Although initially developed for Ordinary Portland Cement (OPC), related theories have since been extended by researchers to account for chemical shrinkage and different curing conditions (saturated vs. sealed). However, as a purely analytical approach, the Powers model yields only global average values and cannot provide spatial distribution information of the pore structure, which limits its ability to analyze local defects.

Currently, various methods are employed to characterize the pore structure of cementitious materials, including Mercury Intrusion Porosimetry (MIP), Nitrogen Adsorption/Desorption (NAD), and Scanning Electron Microscopy (SEM). However, each method has inherent limitations regarding sample preparation, measurement range, or destructiveness. The characteristics and shortcomings of these existing approaches are summarized in Table 1.

In contrast to these traditional techniques, X-ray computed tomography (CT) has emerged as a powerful, non-destructive 3D imaging technique for visualizing the internal microstructure of cement-based materials [8,9], including 3D microcrack analysis [10,11,12,13], pore structure characterization [14,15,16,17,18,19,20], crack propagation identification [20,21] and monitoring of chloride ion ingress in reinforced concrete [22,23]. However, its application faces a critical trade-off between image resolution and the Representative Volume Element (RVE). Nano-CT can achieve sub-micron spatial resolution (hundreds of nm) but is restricted to very small scanned volumes, with typical sample diameters on the order of 0.5–2 mm and volumes around 1 mm^3^ [24,25,26]. Such small volumes are often insufficient to represent the heterogeneous microstructure of a bulk cement paste. Conversely, micro-CT commonly provides voxel sizes from 1 μm up to tens of μm and can image sample sizes from millimeters to several centimeters [27,28,29]. To penetrate these larger volumes, higher X-ray energies are required, which inherently results in a lower effective resolution (typically on the micron scale). The fundamental problem addressed in this research is the inherent conflict between spatial resolution and the Representative Volume Element (RVE) in X-ray CT imaging. While macro-CT can scan large, representative samples necessary for mechanical testing, its resolution is insufficient to detect the nano-scale capillary and gel pores that govern material performance. Consequently, standard segmentation methods applied to macro-CT images systematically fail to capture these fine pores, leading to a severe underestimation of total porosity. There is currently a lack of non-destructive frameworks capable of recovering this ‘invisible’ nano-scale porosity information from macro-scale scans, creating a significant gap between qualitative imaging and quantitative microstructural characterization.

To overcome this fundamental limitation, this study proposes and validates a model-informed framework that ingeniously integrates the Powers’ volume model with micro-CT image analysis. We circumvent the need for nano-scale resolution by leveraging the stoichiometric principles of cement hydration. This approach demonstrates that the total porosity, including the unresolved nano-scale fraction, can be derived by solely segmenting the easily identifiable, micron-scale unhydrated cement phase. This model-informed method transforms CT from a partial characterization tool into a quantitative technique for assessing total porosity.

The primary objective of this work is to establish and verify the feasibility of this integrated approach. We detail the methodology for applying this model to real CT-derived structural data. The accuracy of the derived porosity is validated by correlating the results with established porosity–strength mathematical models. This study confirms that the synergy between the classical Powers’ model and modern CT analysis provides a robust and feasible pathway for the comprehensive microstructural characterization of cementitious materials, bridging a long-standing gap between theory and advanced experimental techniques.

## 2. Materials and Methods

### 2.1. Materials and Specimens

**Figure 1:**
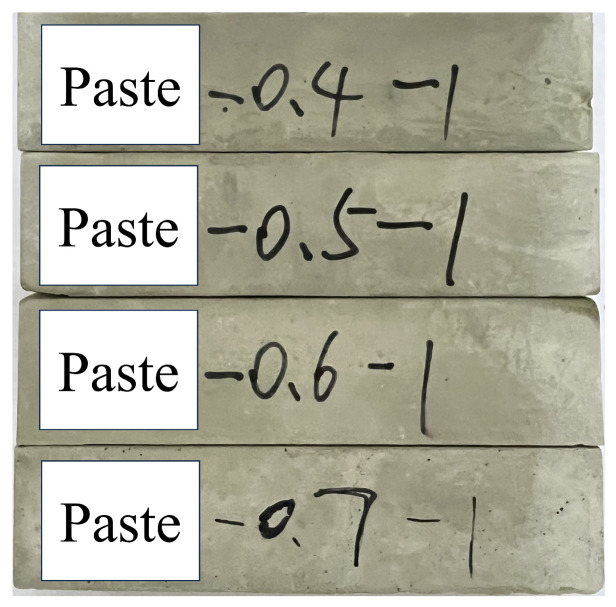
OPC 42.5 cement paste specimens.

The cement used in the experiment was OPC (strength class 42.5) from Hebei Tangshan. The chemical composition of the cement is listed in Table 2. The specimens were molded to dimensions of 40 mm × 40 mm × 160 mm (Figure 1). The *w*/*c* ratios for paste specimens were established at 0.4, 0.5, 0.6, and 0.7. The specimens were demolded at 3 days after molding and subsequently cured in water (saturated system, as explained in Section 2.2.3.) until reaching the 28-day age. Each group consisted of four specimens, three of which were used for mechanical property testing and one for CT analysis.

### 2.2. Methods

#### 2.2.1. Mechanical Properties

After 28 days of curing, the specimens were subjected to flexural and compressive strength tests. A load was applied uniformly at a rate of 50 N/s ± 10 N/s to the opposite side of the prism until fracture occurred. The two halves of the broken prism were kept in a moist state until compressive strength testing. The average value of three specimens was taken as the test result for flexural strength. After completing the flexural strength test, the two halves of each prism were subjected to compressive strength testing. The compressive strength test was performed on the lateral faces of the prism halves. The load was applied uniformly at a rate of 2400 N/s ± 200 N/s until the specimen failed. The average value of six specimens was taken as the test result for compressive strength.

#### 2.2.2. CT Test

**Figure 2:**
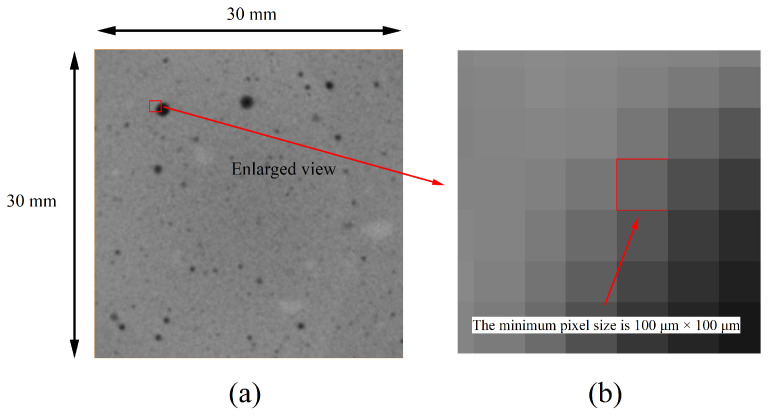
CT image of (**a**) original image and (**b**) enlarged image.

One representative specimen was scanned for each *w*/*c* ratio group. For the CT test, the entire prism specimen (40 mm × 40 mm × 160 mm) was scanned without physical sectioning. X-ray computed tomography (CT) was performed using an industrial high-energy CT system operated at an X-ray tube voltage of 420 kV. The effective voxel size after system calibration was 0.1 mm × 0.1 mm × 0.1 mm. A total of 1650 2D CT images were obtained for each sample and imported into the commercial software Avizo 2022 for 3D reconstruction and image segmentation. To minimize the influence of specimen preparation and edge effects, a digital sampling method was applied to virtually extract the central three-quarters of the specimen’s volume for analysis. The resulting image size is 300 × 300 pixels, with a pixel size of 100 μm × 100 μm (Figure 2). Reconstructed images were preprocessed using a median filter for noise reduction, followed by a ring artifact removal algorithm. To correct for background inhomogeneity caused by beam hardening, a Top-Hat transform was applied. Image segmentation was guided by grayscale histogram analysis. The Otsu algorithm was employed to automatically identify the optimal threshold at the histogram valley, ensuring objective and robust phase separation.

#### 2.2.3. Phase Volume Fraction

**Figure 3:**
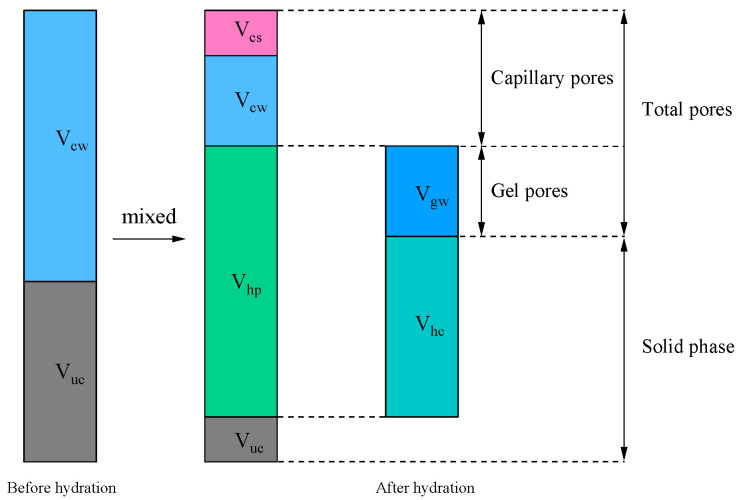
Composition of various phases before and after cement hydration. (Abbreviations: *V*_uc_ is the volume of unreacted cement; *V*_hp_ is the volume of hydration products; *V*_cw_ is the volume of capillary water (unreacted water); *V*_cs_ is the volume of chemical shrinkage; *V*_hc_ is the volume of hydrated cement; *V*_gw_ is the volume of gel water).

**Figure 4:**
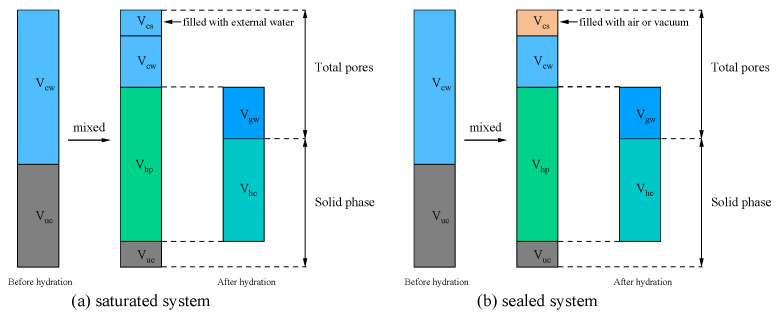
The results of chemical shrinkage volume under different conditions. (Abbreviations: *V*_uc_ is the volume of unreacted cement; *V*_hp_ is the volume of hydration products; *V*_cw_ is the volume of capillary water (unreacted water); *V*_cs_ is the volume of chemical shrinkage; *V*_hc_ is the volume of hydrated cement; *V*_gw_ is the volume of gel water).

Powers and Brownyard [3] proposed a classical cement hydration model (Figure 3). Before hydration occurs, the system consists of unreacted water and cement particles (unhydrated cement). As hydration progresses, part of the cement reacts with water to form hydration products. These products include hydrated cement and gel space, the latter of which may be filled with water (physically bound water). Hydrated cement is composed of the reacted cement and non-evaporable water (chemically bound water). Capillary water, namely the unreacted portion of water, is regarded as “free water”. Chemical shrinkage is an inevitable outcome of cement hydration, as the total volume of hydration products is smaller than the combined volume of the reacted cement and water. The capillary pore volume is not equal to the capillary water volume; in fact, the capillary pore volume of the system is equal to the sum of the capillary water volume and the chemical shrinkage volume. In other words, capillary pores are composed of capillary water and chemical shrinkage. Depending on the conditions, the space generated by chemical shrinkage will exhibit different results (Figure 4). The saturated system corresponds to an open environment (e.g., water curing), where the cement paste is continuously supplied with external water. Consequently, the voids created by chemical shrinkage are replenished and become filled with capillary water. In contrast, the sealed system represents a closed environment (e.g., sealed in molds or wrapped), where no mass exchange with the exterior is permitted. In this case, the chemical shrinkage volume cannot be compensated for by external water and thus remains as empty voids (air or vacuum) within the microstructure.

Building on the research by Powers and Brownyard, Hansen [30], Jensen [31], and Brouwers [32] conducted further studies and discussions on the Powers’ model. However, there are differences in the symbolic representation of the same physical quantity in the research papers of different scholars. Therefore, this paper uses unified symbols for the same phase or definition: for instance, Hansen and Brouwers used the letter “m” to denote the degree of hydration, while Jensen used the letter “*α*”; in this paper, “*α*” is uniformly adopted to represent the degree of hydration. Specifically, Hansen and Brouwers provided a detailed derivation process for the volume fraction of each phase, with the initial *w*/*c* ratio and degree of hydration serving as the core parameters in the derivation. In contrast, Jensen proposed a volume fraction equation with initial porosity and degree of hydration as parameters but did not provide the detailed derivation steps of this equation in his paper. Hansen, Jensen, and Brouwers provided or suggested parameter values in their respective studies, which are summarized in Table 3.

The specific volumes of cement and water (capillary water) are taken as unified values, namely *v*_c_ = 0.32 cm^3^/g and *v*_w_ = 1 cm^3^/g. For the specific volume of non-evaporable water, *v*_n_, Hansen suggested *v*_n_ = 0.75 cm^3^/g whereas Brouwers considered *v*_n_ = 0.72 cm^3^/g more appropriate. Regarding the specific volume of gel water, *v*_g_, Hansen proposed *v*_g_ = 1 cm^3^/g, treating it as uncompressed water; in contrast, Brouwers discussed *v*_g_ = 1 cm^3^/g or *v*_g_ = 0.9 cm^3^/g, and concluded that *v*_g_ = 0.9 cm^3^/g (considered it as compressed water) is more reasonable. Jensen did not provide specific reference values for *v*_n_ and *v*_g_. The parameters *a* and *k* are constant values and can be directly obtained from Hansen’s original reference. For *w*_n_/*c*_h_ and *w*_g_/*c*_h_, which represent the amount of chemically bound water and physically bound water associated with the complete hydration of 1 g of cement, Hansen and Jensen retained the classical results of Powers and Brownyard, namely *w*_n_/*c*_h_ = 0.23 g/g and *w*_g_/*c*_h_ = 0.19 g/g. Although Brouwers discussed these parameters, no definitive values were proposed, and he pointed out that they may vary with cement type. Since this study focuses on ordinary Portland cement, and to ensure consistency with Hansen and Jensen, the values *w*_n_/*c*_h_ = 0.23 g/g and *w*_g_/*c*_h_ = 0.19 g/g were also adopted in Brouwers’ formula, while the specific volume of chemically bound water was uniformly taken as *v*_n_ = 0.75 cm^3^/g. After determining the above parameters, the phase equations proposed by different scholars were calculated, and the results are shown in Table 4.

Since Jensen’s volume fraction equations were derived from the initial porosity, and Brouwers’s equations did not explicitly account for the specific volume of gel water, Hansen’s volume fraction equations were selected in this study. The following presents the volume fraction equations of each phase during the hydration of ordinary Portland cement paste:

(1) $$\varphi_{uc}=\frac{0.32\left(1-\alpha \right)}{\frac{w}{c}+0.32}$$

(2) $$\varphi_{hc}=\frac{0.49\alpha }{\frac{w}{c}+0.32}$$

(3) $$\varphi_{gw}=\frac{0.19\alpha }{\frac{w}{c}+0.32}$$

(4) $$\varphi_{hp}=\frac{0.68\alpha }{\frac{w}{c}+0.32}$$

(5) $$\varphi_{cw}=\frac{\frac{w}{c}-0.42\alpha }{\frac{w}{c}+0.32}$$

(6) $$\varphi_{cs}=\frac{0.06\alpha }{\frac{w}{c}+0.32}$$

(7) $$\varphi_{cp}=\frac{\frac{w}{c}-0.36\alpha }{\frac{w}{c}+0.32}$$

(8) $$\varphi_{tp}=\frac{\frac{w}{c}-0.17\alpha }{\frac{w}{c}+0.32}$$

where *α* is the degree of hydration; *φ*_uc_ is the volume fraction of unhydrated cement; *φ*_hc_ is the volume fraction of hydrated cement; *φ*_gw_ the volume fraction of gel water (gel pores); *φ*_hp_ is the volume fraction of hydration products; *φ*_cw_ is the volume fraction of unreacted capillary water; *φ*_cs_ is the volume fraction of chemical shrinkage; *φ*_cp_ is the volume fraction of capillary pores; *φ*_tp_ is the total porosity.

It is noted that the minimum *w*/*c* ratio necessary to achieve complete hydration differs between systems. In the saturated system [30], the degree of hydration of the cement paste is taken as

(9) $$\alpha ⩽\frac{\frac{w}{c}}{0.36}\;\mathrm{when}\;0<\frac{w}{c}<0.36$$

and

(10) $$\alpha =1\;\mathrm{when}\;\frac{w}{c}⩾0.36$$

In the sealed system [30], the degree of hydration of the cement paste is taken as

(11) $$\alpha ⩽\frac{\frac{w}{c}}{0.42}\;\mathrm{when}\;0<\frac{w}{c}<0.42$$

and

(12) $$\alpha =1\;\mathrm{when}\;\frac{w}{c}⩾0.42$$

In this work, the saturated system is adopted. The volume fractions of unhydrated cement and air pores were automatically calculated by Avizo. However, the Powers’ volume model excludes the air pore phase introduced from the external environment in the process of specimen casting. After obtaining the air pore volume fraction, *φ*_ap_, the theoretical volume fractions of the other phases in the saturated system should be multiplied by a factor of (1 − *φ*_ap_) according to Equations (1)–(8).

#### 2.2.4. The Porosity–Strength Relationship

Porosity is a key factor affecting the compressive strength of cement-based materials. The following four mathematical models are commonly used to predict the relationship between strength and porosity in porous materials [33,34,35,36,37,38,39,40]:

(13) $$\sigma =\sigma_{0}{\left(1-p\right)}^{k}\;(\mathrm{Balshin})$$

(14) $$\sigma =\sigma_{0}e^{-kp}\;(\mathrm{Ryshkewitch})$$

(15) $$\sigma =kln\left(\frac{P_{\sigma 1}}{p}\right)\;(\mathrm{Schiller})$$

(16) $$\sigma =\sigma_{0}-kp\;(\mathrm{Hasselman})$$

where *σ* is the compressive strength; *σ*_0_ is the compressive strength of the material at zero porosity; *k* is an empirical constant; *p* is the total porosity of the material; *p*_σ1_ is the porosity at which the material’s compressive strength becomes 0. In the above equations, the compressive strength at zero porosity (*σ*_0_) and the total porosity corresponding to zero strength (*p*_σ1_) are considered empirical constants obtained from experimental data. After calculating the total porosity of cement paste using the Powers’ volume model, the results can be verified using the aforementioned mathematical models for porous materials.

#### 2.2.5. Statistical and Mathematical Analysis

In this study, quantitative experimental results, particularly for mechanical properties, are presented as mean values ± standard deviation (SD) to characterize the data variance. The error bars in the figures represent the standard deviation derived from replicate measurements (three specimens for flexural strength and six for compressive strength). For the reliability verification of the CT-calculated porosity, the correlation between porosity and compressive strength was analyzed using four classical mathematical models (Balshin, Ryshkewitch, Schiller, and Hasselman). The model parameters were determined using non-linear regression based on the least squares method (Levenberg–Marquardt algorithm). The goodness of fit for each model was evaluated using the coefficient of determination (R^2^) to assess the prediction accuracy and error magnitude.

## 3. Results and Discussion

### 3.1. Analysis of Factors Influencing the Hydration Process

To further analyze the effects of the degree of hydration and the *w*/*c* ratio on the hydration process from Powers’ volume model, the degree of hydration *α* was varied from 0 to 1 in increments of 0.1 and the *w*/*c* ratio ranged from 0.1 (low *w*/*c*) to 0.9 (high *w*/*c*), with particular emphasis on *w*/*c* = 0.36 (the critical value for the saturated system) and *w*/*c* = 0.42 (the critical value for the sealed system), also in increments of 0.1.

#### 3.1.1. Degree of Hydration

The degree of hydration is a critical parameter in cement-based materials, as it governs the volume fractions of different phases in cement paste and consequently determines the strength and durability of the material. This study analyzes the influence of the degree of hydration on phase volume fractions in two systems.

**Figure 5:**
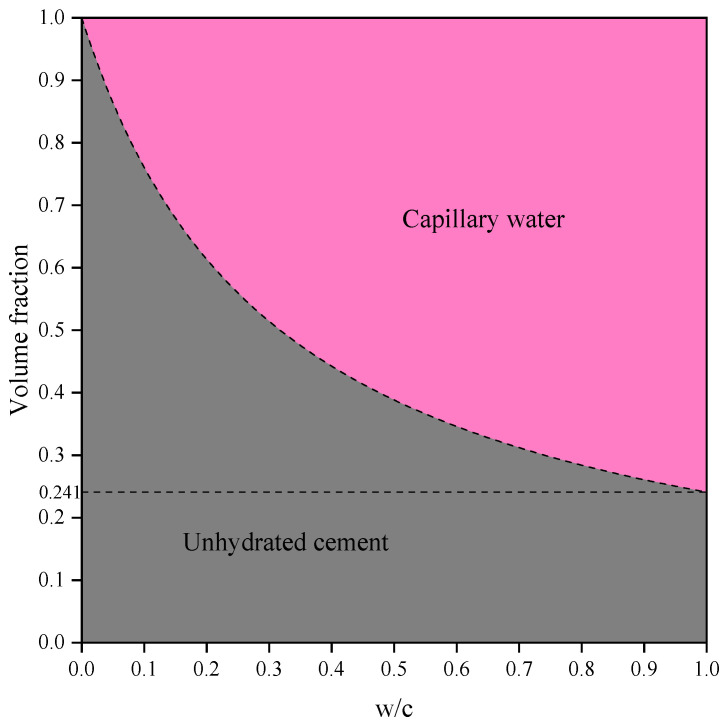
The volume fractions of each phase in cement paste at the initial state (*α* = 0) under saturated and sealed system conditions.

In both saturated and sealed systems, the cement paste exhibits the same initial state (*α* = 0, Figure 5). When *α* = 0 (i.e., immediately after cement and mixing water are combined), only unhydrated cement and capillary water phases exist in the system. At this stage, the initial porosity exhibits a non-linear relationship with *w*/*c*, represented by a hyperbolic curve. For example, when *w*/*c* = 1, the initial porosity of the system is *p*_0_ = 1 − 0.241 = 0.759.

**Figure 6:**
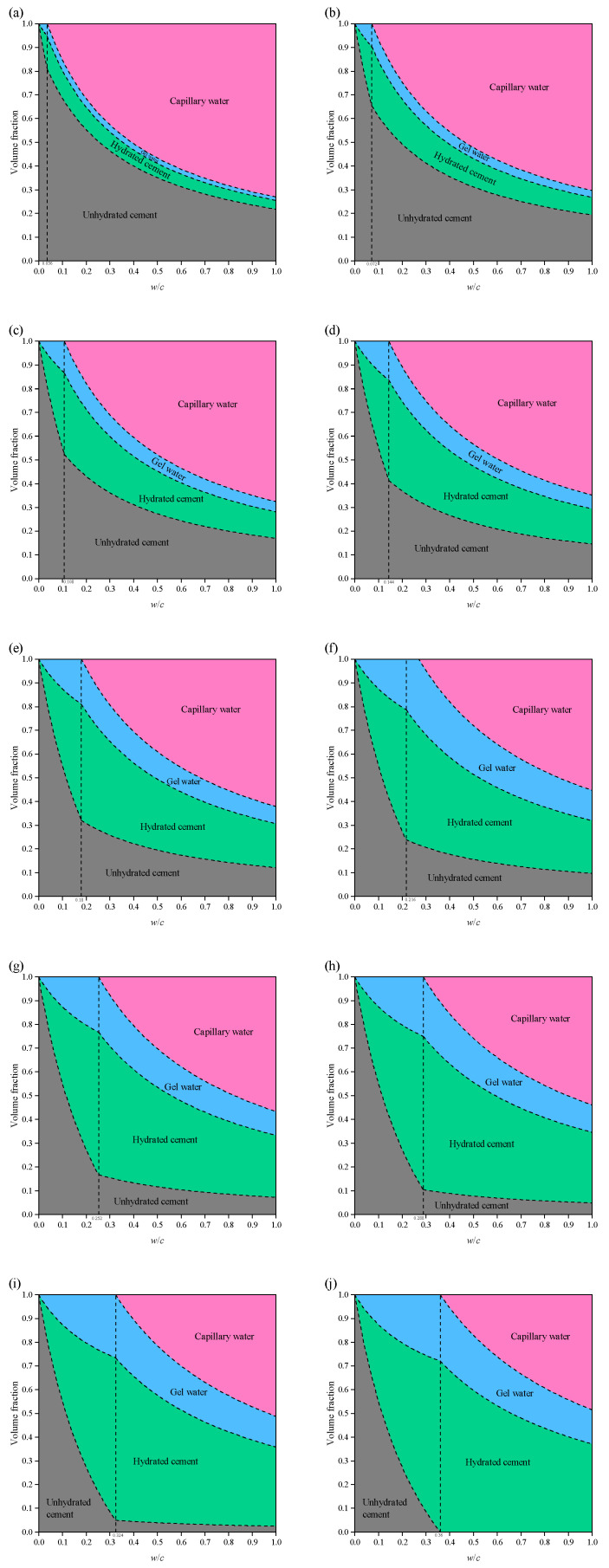
Volume fraction of each phase in cement paste at different degree of hydration in a saturated system: (**a**) *α* = 0.1; (**b**) *α* = 0.2; (**c**) *α* = 0.3; (**d**) *α* = 0.4; (**e**) *α* = 0.5; (**f**) *α* = 0.6; (**g**) *α* = 0.7; (**h**) *α* = 0.8; (**i**) *α* = 0.9; (**j**) *α* = 1.

As shown in Figure 6, the variation in phase volume fractions in saturated system cement paste with *w*/*c* ratio is illustrated as the degree of hydration *α* increases from 0.1 to 1. As hydration progresses, hydration products composed of hydrated cement and gel water gradually appear. A distinct *w*/*c* threshold can be observed in the figure.

Taking *α* = 0.5 as an example: when 0 < *w*/*c* < 0.18, corresponding to the region to the left of the threshold, *φ*_cw_ = 0, indicating that hydration has completely ceased and cannot proceed further. In contrast, when *w*/*c* > 0.18 (right side of the threshold), unhydrated cement and capillary water remain in the system (*φ*_un_ > 0 and *φ*_cw_ > 0), allowing hydration to continue and reach higher degrees of hydration.

When *α* = 1 (ideal state or after sufficient time), for *w*/*c* < 0.36, the cement paste consists only of three phases—unhydrated cement, hydrated cement, and gel water—without the presence of capillary water. In this case, the volume fraction of hydration products increases with *w*/*c* (0 < *w*/*c* < 0.36). At *w*/*c* = 0.36, the system is fully composed of hydration products, with no unhydrated cement or capillary water remaining. For *w*/*c* > 0.36, all cement can be fully hydrated; however, as *w*/*c* continues to increase, the volume fraction of capillary water rises significantly, leading to an increase in total porosity of the cement paste, which in turn has an adverse effect on its strength.

**Figure 7:**
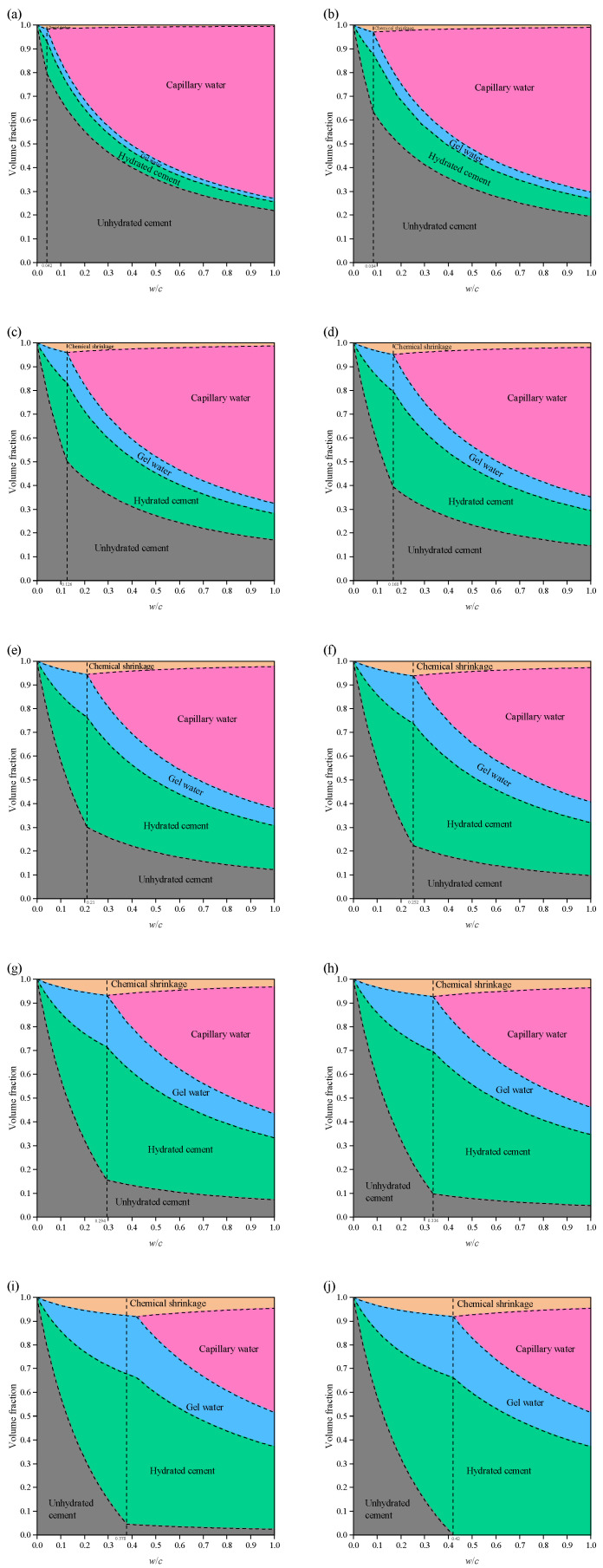
Volume fraction of each phase in cement paste at different degree of hydration in a sealed system: (**a**) *α* = 0.1; (**b**) *α* = 0.2; (**c**) *α* = 0.3; (**d**) *α* = 0.4; (**e**) *α* = 0.5; (**f**) *α* = 0.6; (**g**) *α* = 0.7; (**h**) *α* = 0.8; (**i**) *α* = 0.9; (**j**) *α* = 1.

For sealed systems, the minimum *w*/*c* ratio required for complete hydration is *w*/*c* = 0.42, which differs from that of saturated systems (Figure 7). As hydration progresses, in the absence of an external water supply, the space generated by chemical shrinkage cannot be filled with water and is instead occupied by air or forms a vacuum.

When *α* = 0.5, if 0 < *w*/*c* < 0.21, the cement paste has *φ*_cw_ = 0, and hydration ceases completely, making further reaction impossible. At the same time, compared with the saturated system, the sealed system contains additional voids caused by chemical shrinkage. When *w*/*c* > 0.21, hydration can still proceed. With increasing *w*/*c*, the volume fraction of chemical shrinkage decreases gradually, while the fraction of capillary pores increases correspondingly.

When *α* = 1, if *w*/*c* < 0.42, all capillary water is consumed, and the cement paste consists of unhydrated cement, hydration products, gel water, and chemical shrinkage. When *w*/*c* = 0.42, the system is composed of hydration products and chemical shrinkage, and at this point, the volume fraction of chemical shrinkage reaches its maximum value of *φ*_cs_ = 0.081. Similarly, when *w*/*c* > 0.42, the volume fraction of chemical shrinkage decreases with increasing *w*/*c*, while the fraction of capillary pores increases continuously.

#### 3.1.2. Water/Cement Ratio

**Figure 8:**
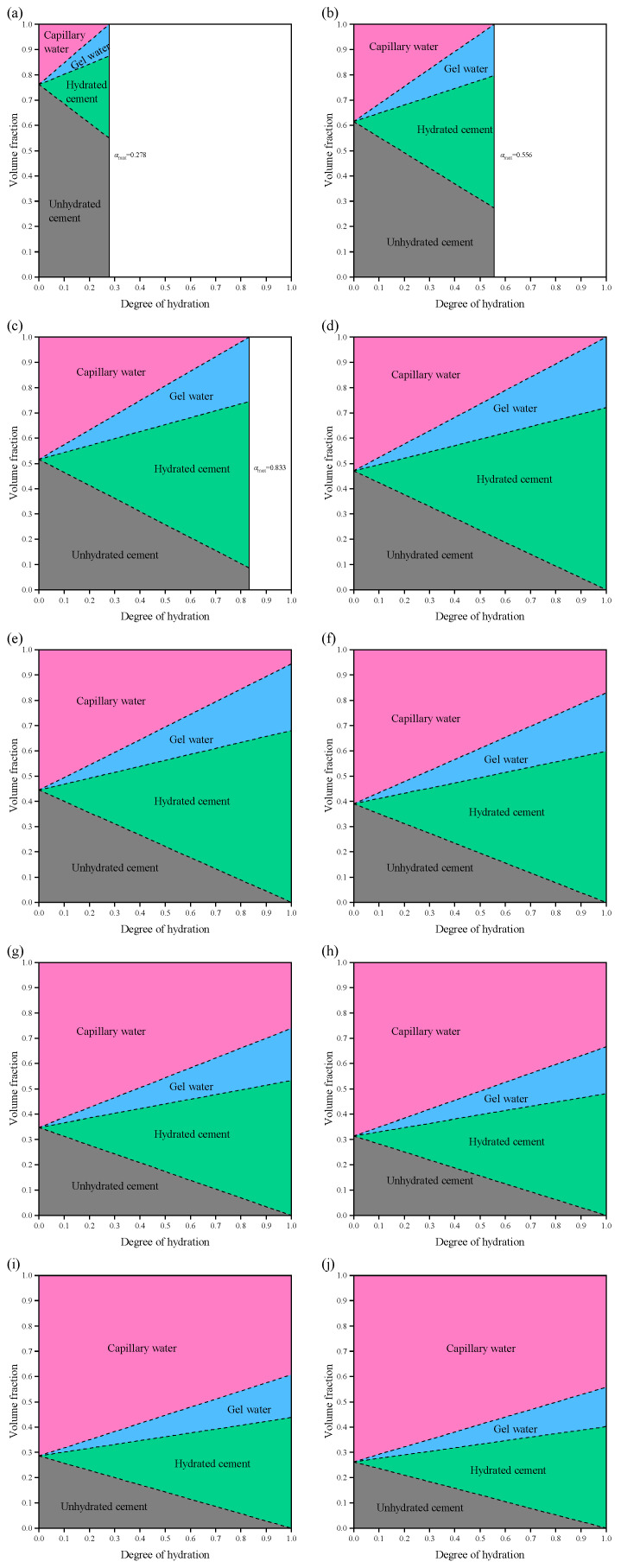
Volume fraction of each phase in cement paste with different *w*/*c* in a saturated system: (**a**) *w*/*c* = 0.1; (**b**) *w*/*c* = 0.2; (**c**) *w*/*c* = 0.3; (**d**) *w*/*c* = 0.36; (**e**) *w*/*c* = 0.4; (**f**) *w*/*c* = 0.5; (**g**) *w*/*c* = 0.6; (**h**) *w*/*c* = 0.7; (**i**) *w*/*c* = 0.8; (**j**) *w*/*c* = 0.9.

Figure 8 illustrates the evolution of the volume fractions of different phases in cement paste within a saturated system, as a function of the degree of hydration (*α*), for *w*/*c* ratios ranging from 0.1 to 0.9. The results indicate that the volume fractions of the phases are linearly related to the degree of hydration. For pastes with *w*/*c* < 0.36 (i.e., *w*/*c* = 0.1, 0.2, and 0.3), it is evident that the maximum degree of hydration is less than 1, implying that complete hydration cannot be achieved. Specifically, the maximum degrees of hydration are *α*_max_ = 0.278 (*w*/*c* = 0.1), *α*_max_ = 0.556 (*w*/*c* = 0.2), and *α*_max_ = 0.833 (*w*/*c* = 0.3), which show a linear relationship *w*/*c*.

A special case occurs at *w*/*c* = 0.36: as hydration progresses, the capillary water is consumed and the unhydrated cement gradually reacts. At *α* = 1, only hydration products remain in the system, and *φ*_hp_ = 1. For cement pastes with *w*/*c* > 0.36, complete hydration can be achieved, but the capillary water fraction increases with increasing *w*/*c*. At *w*/*c* = 0.9 (high *w*/*c* ratio) and *α* = 1, the capillary porosity reaches *φ*_tp_ = 0.598, which significantly reduces the strength of the cement paste. Higher *w*/*c* ratios (*w*/*c* > 1) are unrealistic in practical engineering applications and are therefore not further discussed.

**Figure 9:**
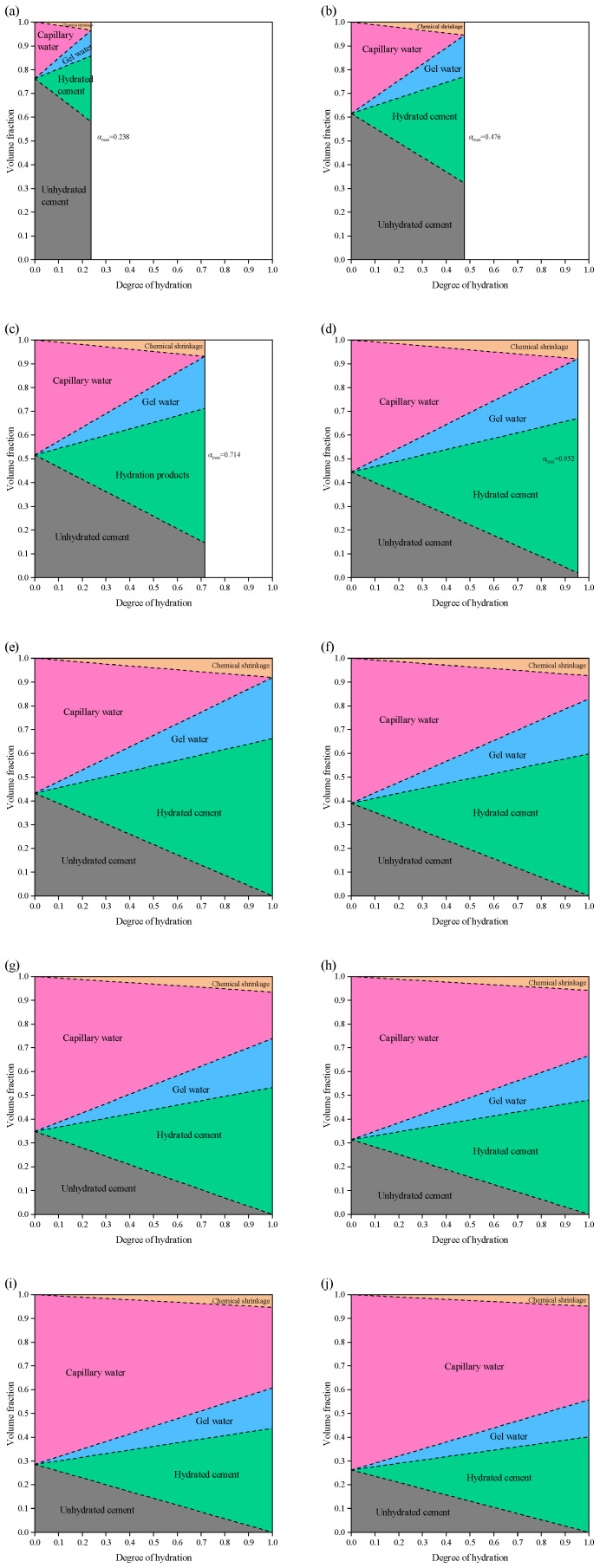
Volume fraction of each phase in cement paste with different *w*/*c* in a sealed system: (**a**) *w*/*c* = 0.1; (**b**) *w*/*c* = 0.2; (**c**) *w*/*c* = 0.3; (**d**) *w*/*c* = 0.4; (**e**) *w*/*c* = 0.42; (**f**) *w*/*c* = 0.5; (**g**) *w*/*c* = 0.6; (**h**) *w*/*c* = 0.7; (**i**) *w*/*c* = 0.8; (**j**) *w*/*c* = 0.9.

Figure 9 presents the evolution of the volume fractions of different phases in cement paste in a sealed system, as a function of the degree of hydration, for *w*/*c* ratios ranging from 0.1 to 0.9. The phase volume fractions also increase linearly with the degree of hydration. When *w*/*c* < 0.42, the maximum degrees of hydration for *w*/*c* = 0.1, 0.2, 0.3, and 0.4 are *α*_max_ = 0.238, 0.476, 0.714, and 0.958, respectively, showing a linear growth trend. At *w*/*c* = 0.42, the cement paste in the system can be fully hydrated (*φ*_uc_ = 0 and *φ*_cw_ = 0). Compared with the saturated system, in addition to hydration products, chemical shrinkage also appears in the sealed system, and the maximum chemical shrinkage volume fraction is *φ*_cs_ = 0.081. For *w*/*c* > 0.42, complete hydration (*α* = 1) can be achieved, and the evolution of unhydrated cement, hydration products, gel water, and capillary water is essentially consistent with that in the saturated system. However, the volume fraction of chemical shrinkage gradually decreases with increasing *w*/*c*.

### 3.2. Flexural and Compressive Strength

**Figure 10:**
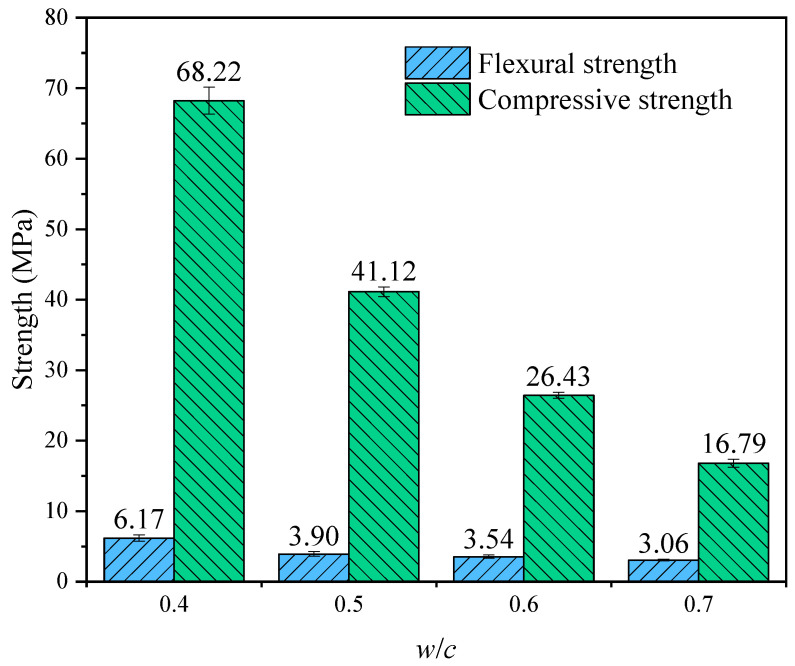
The 28-day flexural and compressive strength of OPC paste with different *w*/*c* ratios.

As illustrated in Figure 10, the *w*/*c* ratio exerts a significant influence on the mechanical properties of the specimens. Generally, both flexural and compressive strengths exhibit a distinct monotonic downward trend as the *w*/*c* increases from 0.4 to 0.7.

Specifically, the compressive strength appears to be highly sensitive to variations in the *w*/*c*. The maximum compressive strength of 68.22 MPa is achieved at a *w*/*c* of 0.4. However, as the *w*/*c* rises to 0.7, the compressive strength experiences a sharp reduction to 16.79 MPa, representing a decrease of approximately 75.4%. Similarly, the flexural strength demonstrates a comparable deterioration pattern, declining from 6.17 MPa (*w*/*c* = 0.4) to 3.06 MPa (*w*/*c* = 0.7). This degradation in mechanical performance can primarily be attributed to the increased porosity and the coarsening of the pore structure within the matrix induced by higher water content, which consequently compromises the compactness and load-bearing capacity of the material.

### 3.3. Image Segmentation

**Figure 11:**
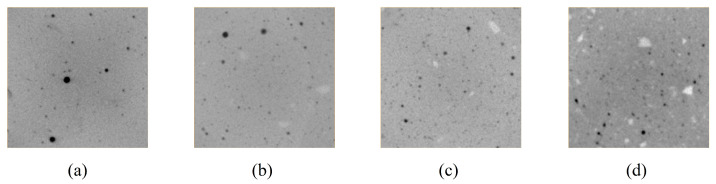
Before threshold segmentation of cement paste CT images: (**a**) *w*/*c* = 0.4; (**b**) *w*/*c* = 0.5; (**c**) *w*/*c* = 0.6; (**d**) *w*/*c* = 0.7.

**Figure 12:**
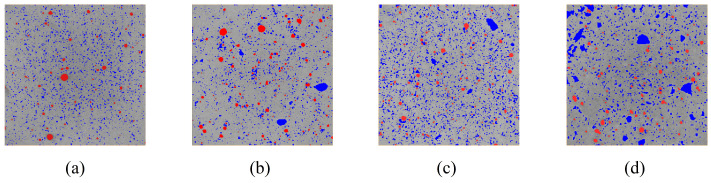
After threshold segmentation (Red represents the air voids and blue indicates unhydrated cement) of cement paste CT images: (**a**) *w*/*c* = 0.4; (**b**) *w*/*c* = 0.5; (**c**) *w*/*c* = 0.6; (**d**) *w*/*c* = 0.7.

After CT testing, 2D images of the cement paste were obtained (Figure 11). To minimize the adverse effects of the edge regions, only three-quarters of the specimen volume was analyzed. The density of hydration products ranges from 2.3 to 2.6 g/cm^3^, while that of unhydrated ordinary Portland cement is 3.15 g/cm^3^. The density of hydration products is lower than that of unhydrated cement. According to the imaging principle of CT, regions with higher density appear brighter. Hence, during image segmentation, the unhydrated cement and pores can be extracted first, and the volume fractions of the remaining phases can then be deduced using the Powers’ volume model. Therefore, Figure 11, the white areas correspond to unhydrated cement, and the black areas represent air pores introduced during specimen molding (not caused by chemical shrinkage). The Interactive Thresholding and Top-Hat functions in Avizo were used for image segmentation to extract the unhydrated cement and air pore phases (Figure 12). It can be observed that in high w/c ratio pastes, unhydrated cement particles are more clustered.

### 3.4. Theoretical Volume Fractions Calculated from Powers’ Model

**Figure 13:**
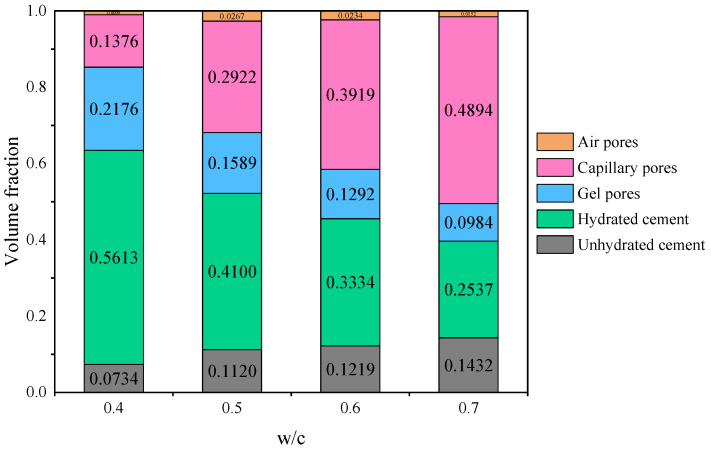
Volume fraction of each phase in cement paste with different *w*/*c* ratios curing for 28 days.

Figure 13 shows the calculated theoretical volume fractions of different phases in cement pastes with *w*/*c* ratios of 0.4, 0.5, 0.6, and 0.7. The results indicate that the air pore volume fraction does not exhibit a clear trend with *w*/*c*, remaining approximately around 3%. In contrast, the other phases show a systematic variation with *w*/*c*. Specifically, the volume fraction of hydrated cement is 0.5613 for the paste with *w*/*c* = 0.4, whereas it decreases to 0.2537 for the paste with *w*/*c* = 0.7, which is less than half of the former. Overall, low *w*/*c* pastes have a higher solid phase fraction and lower porosity, while high *w*/*c* pastes have a lower solid fraction and increased porosity. Notably, the capillary pore volume fraction reaches 0.4894 for the paste with *w*/*c* = 0.7, which is detrimental to the structural strength. Based on the results shown in Figure 13, the total porosity of cement pastes with different *w*/*c* ratios is presented in Table 5. It can be observed that the total porosity of the cement paste increases progressively with increasing *w*/*c* ratio.

### 3.5. Reliability Verification of CT-Calculated Porosity

**Figure 14:**
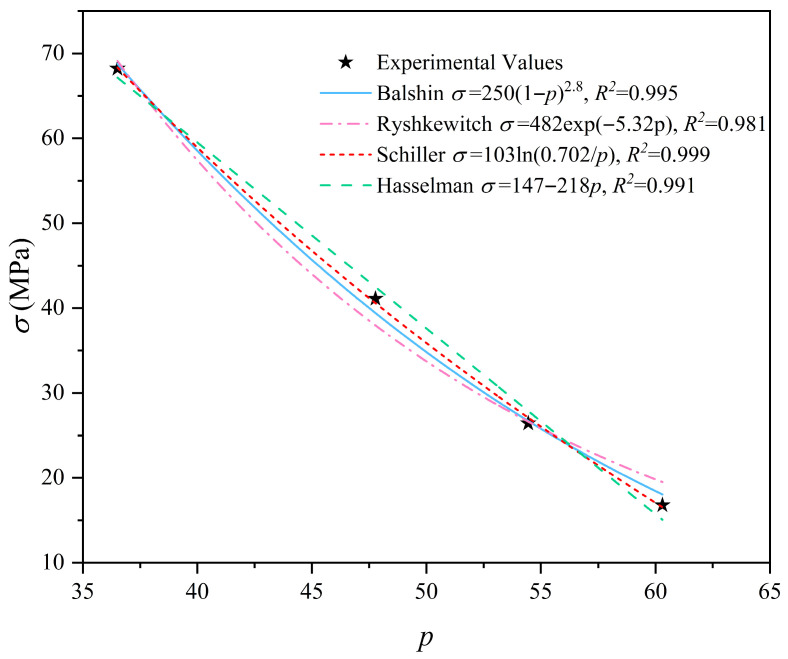
The relationship between the total porosity (%) and strength of cement paste.

Figure 14 illustrates the relationship between the compressive strength and the total porosity calculated by the proposed CT-based method. As expected, the compressive strength shows a clear decreasing trend with the increase in porosity. To verify the reliability of the porosity results, experimental data were fitted using four classical prediction models for porous materials: Balshin, Ryshkewitch, Schiller, and Hasselman. As shown in Figure 14, all four models exhibit excellent agreement with experimental data, with coefficients of determination (*R*^2^) exceeding 0.98. Notably, the Schiller model provides the best fit (*R*^2^ = 0.999), indicating that the relationship between the calculated porosity and strength strictly follows the logarithmic law of porous solids. The high consistency between the experimental values and theoretical models strongly validates the accuracy of the total porosity evaluated by the CT and Powers’ model in this study.

For the same type of cement, the theoretical total porosity of cement paste is identical in saturated and sealed systems when the *w*/*c* ratio and the degree of hydration are the same. The distinction between the two systems lies in the fact that, when the *w*/*c* ratio is lower than 0.42 (the critical value varies with cement type and can be verified according to Brouwers’ conclusions), the saturated system can achieve a higher ultimate degree of hydration under the ideal condition of complete hydration, resulting in a lower total porosity. This is attributed to the facilitation of cement hydration by the availability of external water in the saturated system. Therefore, in this study, the theoretical values of total porosity under the saturated system are adopted as a reference. Considering practical engineering applications, the selected *w*/*c* ratio range is 0.2–0.7. The theoretically calculated total porosity values are listed in Table 6. It should be noted that, in the saturated system, when the *w*/*c* ratios are 0.2 and 0.3, the maximum degrees of hydration are *α*_max_ = 0.556 (*w*/*c* = 0.2) and *α*_max_ = 0.833 (*w*/*c* = 0.3), respectively. These values are not included in the table due to space limitations, and the corresponding total porosities are 0.203 and 0.255, respectively. As shown in Table 6, with decreasing *w*/*c* ratio, the total porosity at the maximum degree of hydration gradually decreases, thereby leading to higher strength.

### 3.6. Limitations of the Proposed Framework

While the proposed framework successfully quantifies total porosity, certain limitations exist regarding both experimental precision and theoretical applicability. First, the accuracy relies fundamentally on the precise segmentation of the unhydrated cement phase. Consequently, experimental factors such as image noise and the inherent resolution limits of the CT system could introduce uncertainties in identifying fine particles. Second, the current model parameters are specific to OPC; systems containing supplementary cementitious materials (e.g., fly ash) would require modified stoichiometric coefficients. Future work will focus on extending this framework to blended cement systems and conducting comparative analyses with other pore characterization methods (e.g., MIP and TGA) to further cross-validate the results.

## 4. Conclusions

This study successfully developed and validated a novel framework that integrates the classical Powers’ hydration model with micro-computed tomography (micro-CT) analysis to achieve a comprehensive and quantitative characterization of the total porosity in cement pastes. The principal findings and their implications are summarized as follows:

1. A model-informed framework was established to quantify the total porosity of cement pastes by integrating micro-CT segmentation of unhydrated cement with the Powers’ volume model, effectively overcoming the resolution limits of conventional CT.
2. The integrated method provides results consistent with established material science principles. The total porosity values calculated using this framework showed a strong correlation (R^2^ > 0.98) with the mechanical strength of laboratory specimens when evaluated against well-established porosity–strength models. This validation confirms the physical realism and accuracy of the proposed method.
3. By enabling the assessment of nano-scale features using micrometer-scale imaging parameters, the integrated method significantly extends the quantitative capabilities of micro-CT without the need for high-energy radiation sources or nano-scale CT systems.

## Figures and Tables

**Table 1:** Comparison of existing methods for characterizing cement pore structure.

Method	Principle	Advantages	Limitations
MIP	Pressurizing mercury into pores	Wide measurement range	Destructive; “Ink-bottle” effect misinterprets pore size; Hazardous material
NAD	Gas condensation in pores	Effective for micro/mesopores (<50 nm)	Limited to surface analysis; Complex sample preparation; Cannot measure macropores
SEM	Electron imaging	High resolution (nm-scale); 2D morphology	2D only; Small field of view; High vacuum required (may alter hydrates)
Micro-CT	X-ray attenuation	Non-destructive; 3D visualization; Macro-scale coverage	Resolution limit (microns): Cannot resolve nano-scale gel pores, leading to underestimated porosity

**Table 2:** Chemical composition (%) of cement experimental materials.

Ingredient	CaO	SiO_2_	Al_2_O_3_	SO_3_	Fe_2_O_3_	MgO	TiO_2_	LOI
OPC	56.25	22.98	7.01	4.23	2.84	4.22	0.67	1.15

**Table 3:** Parameters provided by different authors.

Author	*v*_c_ (cm^3^/g)	*v*_w_ (cm^3^/g)	*v*_n_(cm^3^/g)	*v*_g_ (cm^3^/g)	*a*	*k*	*w*_n_/*c*_h_	*w*_g_/*c*_h_
Hansen [30]	0.32	1	0.75	1	3.3	0.25	0.23	0.19
Jensen [31]	0.32	1					0.23	0.19
Brouwers [32]	0.32	1	0.72	1 or 0.9				

Note: *v*_c_ is the specific volume of cement; *v*_w_ is the specific volume of water; *v*_n_ is the specific volume of non-evaporable water; *v*_g_ is the specific volume of gel water; *a* is a constant with a value of approximately 3.3; *k* is a constant with a value of approximately 0.25; *w_n_*/*c_h_* is the chemically bound water with the complete hydration of 1 g of cement; *w_g_*/*c_h_* is the physically bound water with the complete hydration of 1 g of cement.

**Table 4:** Volume fraction equations of different phases as provided by various researchers.

Phase	Hansen	Jensen	Brouwers	
			*v*_g_ = 1 cm^3^/g	*v*_g_ = 0.9 cm^3^/g (Suggested with Brouwers)
*φ*_uc_	$\frac{\left(1-\alpha \right)v_{c}}{\frac{w}{c}+v_{c}}=\frac{0.32\left(1-\alpha \right)}{\frac{w}{c}+0.32}$	$\frac{\left(1-\alpha \right)v_{c}}{\frac{w}{c}+v_{c}}=\frac{0.32\left(1-\alpha \right)}{\frac{w}{c}+0.32}$	$\frac{\left(1-\alpha \right)v_{c}}{\frac{w}{c}+v_{c}}=\frac{0.32\left(1-\alpha \right)}{\frac{w}{c}+0.32}$	$\frac{\left(1-\alpha \right)v_{c}}{\frac{w}{c}+v_{c}}=\frac{0.32\left(1-\alpha \right)}{\frac{w}{c}+0.32}$
*φ*_hc_	$\frac{\left(v_{c}+v_{n}\frac{w_{n}}{c_{h}}\right)\alpha }{\frac{w}{c}+v_{c}}=\frac{0.4925\alpha }{\frac{w}{c}+0.32}$	$\frac{1.52v_{c}\alpha }{\frac{w}{c}+v_{c}}=\frac{0.4864\alpha }{\frac{w}{c}+0.32}$	$\frac{\left(\frac{v_{c}}{v_{w}}+\frac{v_{n}}{v_{w}}\frac{w_{n}}{c_{h}}\right)\alpha }{\frac{w}{c}+\frac{v_{c}}{v_{w}}}=\frac{0.4856\alpha }{\frac{w}{c}+0.32}$	$\frac{\left(\frac{v_{c}}{v_{w}}+\frac{v_{n}}{v_{w}}\frac{w_{n}}{c_{h}}\right)\alpha }{\frac{w}{c}+\frac{v_{c}}{v_{w}}}=\frac{0.4856\alpha }{\frac{w}{c}+0.32}$
*φ*_gw_	$\frac{ak\alpha \frac{w_{n}}{c_{h}}}{\frac{w}{c}+v_{c}}=\frac{0.18975\alpha }{\frac{w}{c}+0.32}$	$\frac{0.60v_{c}\alpha }{\frac{w}{c}+v_{c}}=\frac{0.192\alpha }{\frac{w}{c}+0.32}$	$\frac{\alpha \frac{v_{g}}{v_{w}}\frac{w_{g}}{c_{h}}}{\frac{w}{c}+\frac{v_{c}}{v_{w}}}=\frac{0.19\alpha }{\frac{w}{c}+\frac{v_{c}}{v_{w}}}$	$\frac{\alpha \frac{v_{g}}{v_{w}}\frac{w_{g}}{c_{h}}}{\frac{w}{c}+\frac{v_{c}}{v_{w}}}=\frac{0.171\alpha }{\frac{w}{c}+\frac{v_{c}}{v_{w}}}$
*φ*_hp_	$\frac{\alpha v_{c}+\left(v_{n}+ak\right)\alpha \frac{w_{n}}{c_{h}}}{\frac{w}{c}+v_{c}}=\frac{0.68225\alpha }{\frac{w}{c}+0.32}$	$\frac{2.12v_{c}\alpha }{\frac{w}{c}+v_{c}}=\frac{0.6784\alpha }{\frac{w}{c}+v_{c}}$	$\frac{\left(\frac{v_{c}}{v_{w}}+\frac{v_{d}}{v_{w}}\frac{w_{d}}{c_{h}}\right)\alpha }{\frac{w}{c}+\frac{v_{c}}{v_{w}}}=\frac{0.6756\alpha }{\frac{w}{c}+0.32}$	$\frac{\left(\frac{v_{c}}{v_{w}}+\frac{v_{d}}{v_{w}}\frac{w_{d}}{c_{h}}\right)\alpha }{\frac{w}{c}+\frac{v_{c}}{v_{w}}}=\frac{0.6566\alpha }{\frac{w}{c}+0.32}$
*φ*_cw_	$\frac{\frac{w}{c}-\left(1+ak\right)\alpha \frac{w_{n}}{c_{h}}}{\frac{w}{c}+v_{c}}=\frac{\frac{w}{c}-0.41975\alpha }{\frac{w}{c}+0.32}$	$\frac{\frac{w}{c}-1.32v_{c}\alpha }{\frac{w}{c}+v_{c}}=\frac{\frac{w}{c}-0.4224\alpha }{\frac{w}{c}+0.32}$	$\frac{\frac{w}{c}-\alpha \frac{w_{d}}{c_{h}}}{\frac{w}{c}+\frac{v_{c}}{v_{w}}}=\frac{\frac{w}{c}-0.42\alpha }{\frac{w}{c}+\frac{v_{c}}{v_{w}}}$	$\frac{\frac{w}{c}-\alpha \frac{w_{d}}{c_{h}}}{\frac{w}{c}+\frac{v_{c}}{v_{w}}}=\frac{\frac{w}{c}-0.42\alpha }{\frac{w}{c}+\frac{v_{c}}{v_{w}}}$
*φ*_cs_	$\frac{\left(1-v_{n}\right)\alpha \frac{w_{n}}{c_{h}}}{\frac{w}{c}+v_{c}}=\frac{0.0575\alpha }{\frac{w}{c}+0.32}$	$\frac{0.20v_{c}\alpha }{\frac{w}{c}+v_{c}}=\frac{0.064\alpha }{\frac{w}{c}+0.32}$	$\frac{\left(\frac{w_{d}}{c_{h}}-\frac{v_{d}}{v_{w}}\frac{w_{d}}{c_{h}}\right)\alpha }{\frac{w}{c}+\frac{v_{c}}{v_{w}}}=\frac{0.0644\alpha }{\frac{w}{c}+\frac{v_{c}}{v_{w}}}$	$\frac{\left(\frac{w_{d}}{c_{h}}-\frac{v_{d}}{v_{w}}\frac{w_{d}}{c_{h}}\right)\alpha }{\frac{w}{c}+\frac{v_{c}}{v_{w}}}=\frac{0.0834\alpha }{\frac{w}{c}+\frac{v_{c}}{v_{w}}}$
*φ*_cp_	$\frac{\frac{w}{c}-\left(v_{n}+ak\right)\alpha \frac{w_{n}}{c_{h}}}{\frac{w}{c}+v_{c}}=\frac{\frac{w}{c}-0.36225\alpha }{\frac{w}{c}+0.32}$	$\frac{\frac{w}{c}-1.12v_{c}\alpha }{\frac{w}{c}+v_{c}}=\frac{\frac{w}{c}-0.3584\alpha }{\frac{w}{c}+v_{c}}$	$\frac{\frac{w}{c}-\alpha \frac{v_{d}}{v_{w}}\frac{w_{d}}{c_{h}}}{\frac{w}{c}+\frac{v_{c}}{v_{w}}}=\frac{0.32-0.3556\alpha }{\frac{w}{c}+0.32}$	$\frac{\frac{w}{c}-\alpha \frac{v_{d}}{v_{w}}\frac{w_{d}}{c_{h}}}{\frac{w}{c}+\frac{v_{c}}{v_{w}}}=\frac{0.32-0.3366\alpha }{\frac{w}{c}+0.32}$
*φ*_tp_	$\frac{\frac{w}{c}-v_{n}\alpha \frac{w_{n}}{c_{h}}}{\frac{w}{c}+v_{c}}=\frac{\frac{w}{c}-0.1725\alpha }{\frac{w}{c}+v_{c}}$	$\frac{\frac{w}{c}-0.62v_{c}\alpha }{\frac{w}{c}+v_{c}}=\frac{\frac{w}{c}-0.1984\alpha }{\frac{w}{c}+v_{c}}$	$\frac{\frac{w}{c}-\alpha \frac{v_{n}}{v_{w}}\frac{w_{n}}{c_{h}}}{\frac{w}{c}+\frac{v_{c}}{v_{w}}}=\frac{\frac{w}{c}-0.1656\alpha }{\frac{w}{c}+\frac{v_{c}}{v_{w}}}$	$\frac{\frac{w}{c}-\alpha \frac{v_{n}}{v_{w}}\frac{w_{n}}{c_{h}}}{\frac{w}{c}+\frac{v_{c}}{v_{w}}}=\frac{\frac{w}{c}-0.1656\alpha }{\frac{w}{c}+\frac{v_{c}}{v_{w}}}$

Note: *w*_d_ is the mass of compressed water and *w*_g_ is the mass of gel water.

**Table 5:** Total porosity of cement pastes with different *w*/*c* ratios calculated using the Powers’ volume model.

*w*/*c* = 0.4	*w*/*c* = 0.5	*w*/*c* = 0.6	*w*/*c* = 0.7
0.3651	0.4778	0.5445	0.6030

**Table 6:** Theoretical total porosity of cement pastes for ordinary Portland cement with different *w*/*c* ratios at degrees of hydration from 0 to 1 in the saturated system.

*w*/*c*	*α* = 0	*α* = 0.1	*α* = 0.2	*α* = 0.3	*α* = 0.4	*α* = 0.5	*α* = 0.6	*α* = 0.7	*α* = 0.8	*α* = 0.9	*α* = 1
0.2	0.385	0.352	0.319	0.287	0.254	0.221					
0.3	0.484	0.456	0.429	0.402	0.374	0.347	0.319	0.292	0.255		
0.36	0.529	0.504	0.479	0.454	0.429	0.404	0.379	0.354	0.329	0.304	0.279
0.4	0.556	0.532	0.508	0.485	0.461	0.438	0.414	0.390	0.367	0.343	0.319
0.5	0.610	0.589	0.568	0.548	0.527	0.506	0.485	0.465	0.444	0.423	0.402
0.6	0.652	0.634	0.615	0.597	0.578	0.560	0.541	0.523	0.504	0.486	0.467
0.7	0.686	0.670	0.653	0.636	0.620	0.603	0.586	0.570	0.553	0.536	0.520

## Data Availability

The original contributions presented in this study are included in the article. Further inquiries can be directed to the corresponding authors.

## Abbreviations

Roman	
*a*	A constant
*c*	Initial water mass
*k*	A constant
*p*	Porosity
*w*	Initial mass of unreacted cement
*w*/*c*	Water/cement ratio
*V*	Volume of different phases
Greek	
*α*	Degree of hydration
*v*	Specific volume [cm^3^/g]
*ρ*	Density [g/cm^3^]
*σ*	Compressive strength
*φ*	Volume fraction in cement paste
Subscript	
0	Initial state or value is 0
*c*	Cement
*cp*	Capillary pores
*cs*	Chemical shrinkage
*cw*	Capillary water
*d*	Compressed water
*gw*	Gel water
*h*	Hydrated
*hc*	Hydrated cement
*hp*	Hydration products
*max*	Maximum
*n*	Nonevaporable
*tp*	Total pores
*uc*	Unhydrated cement
*w*	Water
*w_n_/c_h_*	Chemically bound water with the complete hydration of 1 g of cement [g/g]
*w_g_/c_h_*	Physically bound water with the complete hydration of 1 g of cement [g/g]
*σ_0_*	Material’s compressive strength becomes 0
